# Hantaviruses and the dilution effect in Southeast Asia

**Published:** 2011-01-10

**Authors:** KR Blasdell, S Morand, Y Chaval, V Herbreteau, B Douangboupha, S Jittapalapong, JF Cosson, P Buchy

**Affiliations:** 1 Institut Pasteur du Cambodge, Unité de Virologie, Phnom Penh, Cambodia; 2 Institut des Sciences de l'Evolution, CNRS, IRD, Université Montpellier 2, 34095 Montpellier, France; 3 Centre de Biologie et de Gestion et des Populations (CBGP), International de Baillarguet, 34988 Montferrier sur lez, France; 4 CIRAD, UR AGIRs (Animal et Gestion Intégrée des Risques), Campus International de Baillarguet, Montpellier, France; 5 National Agricultural Research Centre, National Agricultural and Forestry Research Institute, Vientiane, Lao PDR; 6 Department of Parasitology, Faculty of Veterinary Medicine, Kasetsart University, Bangkock 10900, Thailand; 7 INRA, UMR CBGP (INRA/IRD/CIRAD/Montpellier SupAgro), Campus international de Baillarguet, 34988 Montferrier sur Lez, France

## Background

Until recently hantaviruses were poorly studied in Southeast Asian countries. To better understand the ecology of hantaviruses in Southeast Asia, we conducted a large scale serological survey of rodent species in several locations in Cambodia, Laos and Thailand. Recent articles indicated that the dilution effect (whereby pathogen prevalence increases with decreasing biodiversity) applied to some American hantavirus species. Therefore we also analyzed our data to establish if any relationship existed between hantavirus seroprevalence and rodent species diversity.

## Methods

Small mammals were live-trapped in different habitat types within a 20 km^2^ radius at seven main locations. Morphological and genetic criteria were employed to identify rodent species. An indirect immuno-fluorescence test was used to detect anti-hantaviruses IgG antibodies in sera. Rodent communities were characterized by species richness estimators utilizing several different indices. Diversity was then estimated using the reciprocal Simpson index and relationships between rodent diversities and hantavirus prevalence was investigated with the Kendall's rank correlation.

## Results

Seropositive rodents were detected at five sites, with prevalence varying from 0 to 5.13%. Antibodies were detected in several rodent species (*Rattus exulans, Rattus nitidius, Rattus norvegicus, Rattus tanezumi, Maxomys surifer, Bandicota indica, Bandicota savelei*, *Mus cookii* and *Mus caroli*). The species prevalence at each site varied between 0-50%. Seropositive animals were more commonly found at sites with lower rodent biodiversity.

## Conclusion

This study further increases the knowledge regarding hantavirus presence in southeast Asia. Seroprevalence rates observed were comparable to those found in previous studies. Because hantavirus seroprevalence increases as rodent biodiversity decreases, the risk for human disease may also potentially increase in southeast Asian areas where habitats are undergoing large-scale modifications with subsequent reduction in natural biodiversity.

